# Retrospective analysis of 51 intralesionally treated cases with progressed giant cell tumor of the bone: local adjuvant use of hydrogen peroxide reduces the risk for tumor recurrence

**DOI:** 10.1186/s12957-019-1613-9

**Published:** 2019-04-23

**Authors:** Georg W. Omlor, Jessica Lange, Marcus Streit, Simone Gantz, Christian Merle, Thomas Germann, Gunhild Mechtersheimer, Jörg Fellenberg, Burkhard Lehner

**Affiliations:** 1 Center of Orthopaedics, Trauma Surgery and Paraplegiology, Heidelberg University Hospital, Schlierbacher Landstrasse 200a, 69118 Heidelberg, Germany; 2 Institute of Pathology Heidelberg, University of Heidelberg, 69120 Heidelberg, Germany; 3 Department of Diagnostic and Interventional Radiology, University of Heidelberg, 69120 Heidelberg, Germany

**Keywords:** Giant cell tumor, Intralesional resection, Curettage, Bone cement, Hydrogen peroxide

## Abstract

**Background:**

Giant cell tumor of the bone (GCT) has high local recurrence rates and the prognosis is hard to predict. We therefore retrospectively analyzed clinical outcome and recurrences of 51 GCT cases focusing on the effects of adjuvant local use of hydrogen peroxide.

**Methods:**

The series enclosed 51 advanced GCT cases of the upper and lower extremities (*n* = 27 Campanacci grade III; *n* = 24 grade II; *n* = 39 surgery at our institution, *n* = 12 elsewhere). Mean follow-up was 88.3 (± 62.0) months. Surgical details, histology, metastases, recurrences, and interview-based data on satisfaction and function including the Musculoskeletal Tumor Society (MSTS) score were evaluated. It was investigated whether hydrogen peroxide was additionally used or not to clean the tumor cavity after curettage as we hypothesized influence on recurrences. To analyze the underlying mechanisms, GCT-derived stromal cell lines were cultured in vitro and tested for cell viability and apoptosis after treatment with hydrogen peroxide. Statistical analysis was performed with Student’s *t* tests, analysis of variance (ANOVA) with post hoc testing, Mann-Whitney *U* tests, chi-square tests, Kaplan-Meier analysis, and multivariate Cox regression analysis.

**Results:**

The whole series had 21 recurrences (41%). Eleven recurrences were found (28%) after surgery at our institution. Kaplan-Meier analysis of cumulative recurrence-free survival revealed at 2 years follow-up 69% (72%, only our institution) and at 10 years follow-up 54% (68%, only our institution). Intralesional resection was performed by vigorous curettage, burring, and defect filling with either polymethylmethacrylate bone cement (*n* = 45) or cancellous bone from the iliac crest (*n* = 6). Univariate chi-square analysis showed significantly lower recurrence rate after bone cement filling (2.3-fold, *p* = 0.024). Cleaning of the lesion cavity with hydrogen peroxide significantly reduced recurrence rate (whole collective 2.9-fold, *p* = 0.004; our institution 2.8-fold, *p* = 0.04) and significantly increased cumulative recurrence-free survival rate (whole collective at 10 years follow-up 74% versus 31%, *p* = 0.002; our institution 79% versus 48%, *p* = 0.02) compared to cases without hydrogen peroxide treatment. In multivariate analysis, significant risk factors for recurrence were pathological fracture (hazard ratio 3.7; *p* = 0.04), high mitosis rate (hazard ratio 15.6; *p* = 0.01), and lack of hydrogen peroxide use (hazard ratio 6.0; *p* = 0.02). In vitro cell culture analyses found apoptotic nature of hydrogen peroxide induced GCT cell death.

**Conclusions:**

The present series proved for the first time that additional cleaning of the tumor cavity with hydrogen peroxide before defect filling significantly reduced recurrence rate and significantly increased recurrence-free survival in advanced but intralesionally treated GCT cases.

## Background

Giant cell tumor of the bone (GCT) is a challenging orthopedic disease representing 10–15% of all benign and 4–5% of all primary bone tumors [1]. GCT has unpredictable biological behavior with locally destructive growth, frequent local recurrences, multifaceted histological appearance, and potential of pulmonary metastases [2–4]. Sixty to eighty percent of all GCT are found in the third and fourth decade of life. Women have a higher risk than men [5]. At the time of initial diagnosis, pathological fracture with clinical symptoms of instability is often already evident [2, 6]. Systemic therapy with denosumab has been introduced as a new encouraging tool to limit activity and growth of GCT but has also been discussed controversially because of unclear benefits and risks [4, 7–9]. Surgical treatment remains the gold standard but new surgical treatment strategies have not been established for a long time, and the literature reports highly heterogeneous study results with inconsistent data [5, 7–13]. Avoiding local recurrence and achieving the best functional outcome remains a hard challenge. Despite recurrence rates up to 65%, intralesional resection with joint preservation and vigorous curettage is most often preferred due to less morbidity compared to extralesional resection [3, 5, 14–16]. Most authors prefer polymethylmethacrylate (PMMA) bone cement filling after curettage instead of cancellous bone, but evidence is inconsistent [13–21]. Cell-toxic local adjuncts such as phenol can reduce the risk for recurrence [22]. Multiple other local adjuvants have been described such as liquid nitrogen, alcohol, iodine, cyclophosphamide, and especially hydrogen peroxide, but due to their heterogeneous use, the effectiveness on recurrence rates and recurrence-free survival has never been proven for one of these individual substances, so far [5, 18, 22, 23].

The aim of the study was to retrospectively analyze recurrence rates, cumulative recurrence-free survival rates, metastases, complications, and functionality and satisfaction after surgical treatment of progressed but intralesionally treated GCT cases. As a novelty of this study, we focused on the additional effect of adjuvant local hydrogen peroxide treatment. For the first time, the present study presents proof of principle in a clinically relevant in vitro analysis of hydrogen peroxide effects on GCT cells together with retrospective clinical data of progressed GCT cases either treated with hydrogen peroxide or not.

## Methods

### Retrospective analysis of surgical outcome

We included all benign GCT cases of the upper and lower extremities followed in our musculoskeletal oncology outpatient clinic from 2000 to 2017 (level I bone and soft tissue tumor center and orthopedic and trauma surgery university hospital). Approval was given by our local ethical committee. Multilocular GCT, lesions of the pelvis and spine, cases with follow-up less than 6 months, and cases without sufficient information regarding H_2_O_2_ application in the surgical reports were excluded. The final sample size was 51 patients, including 39 patients with first surgical treatment at our institution and 12 patients with first surgery elsewhere. Mean follow-up was 88.3 (± 62.0) months. Minimal follow-up was 7 months. Mean patient age was 38.7 (± 13.3) years, *n* = 26 were male, and *n* = 25 were female. No patient was lost to follow-up.

**Fig. 1:**
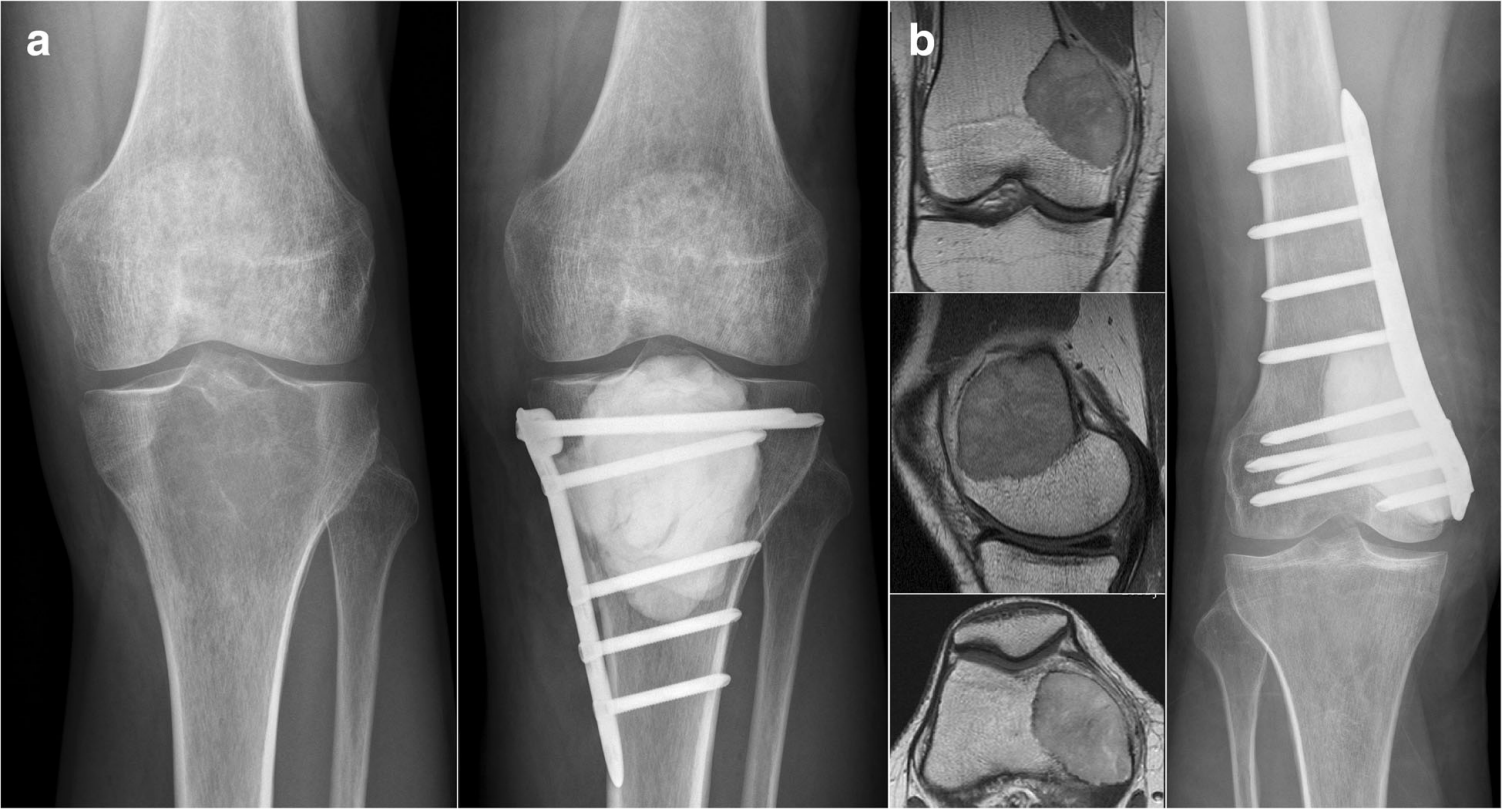
Intralesional resection strategy with bone cement filling and additional osteosynthesis at the proximal tibia (**a**) and distal femur (**b**) with preoperative images (on the left) and postoperative images (on the right)

Diagnosis was verified by open incision biopsy. Surgical strategy was intralesional resection with aggressive curettage. After curettage, the cavity was most often filled with polymethylmethacrylate bone cement (*n* = 45; Fig. 1; Palacos® R+G; Heraeus Medical, Hanau, Germany) and less frequently with cancellous bone from the iliac crest (*n* = 6). The upper extremity was affected in *n* = 9 and the lower extremity in *n* = 42 cases.

**Fig. 2:**
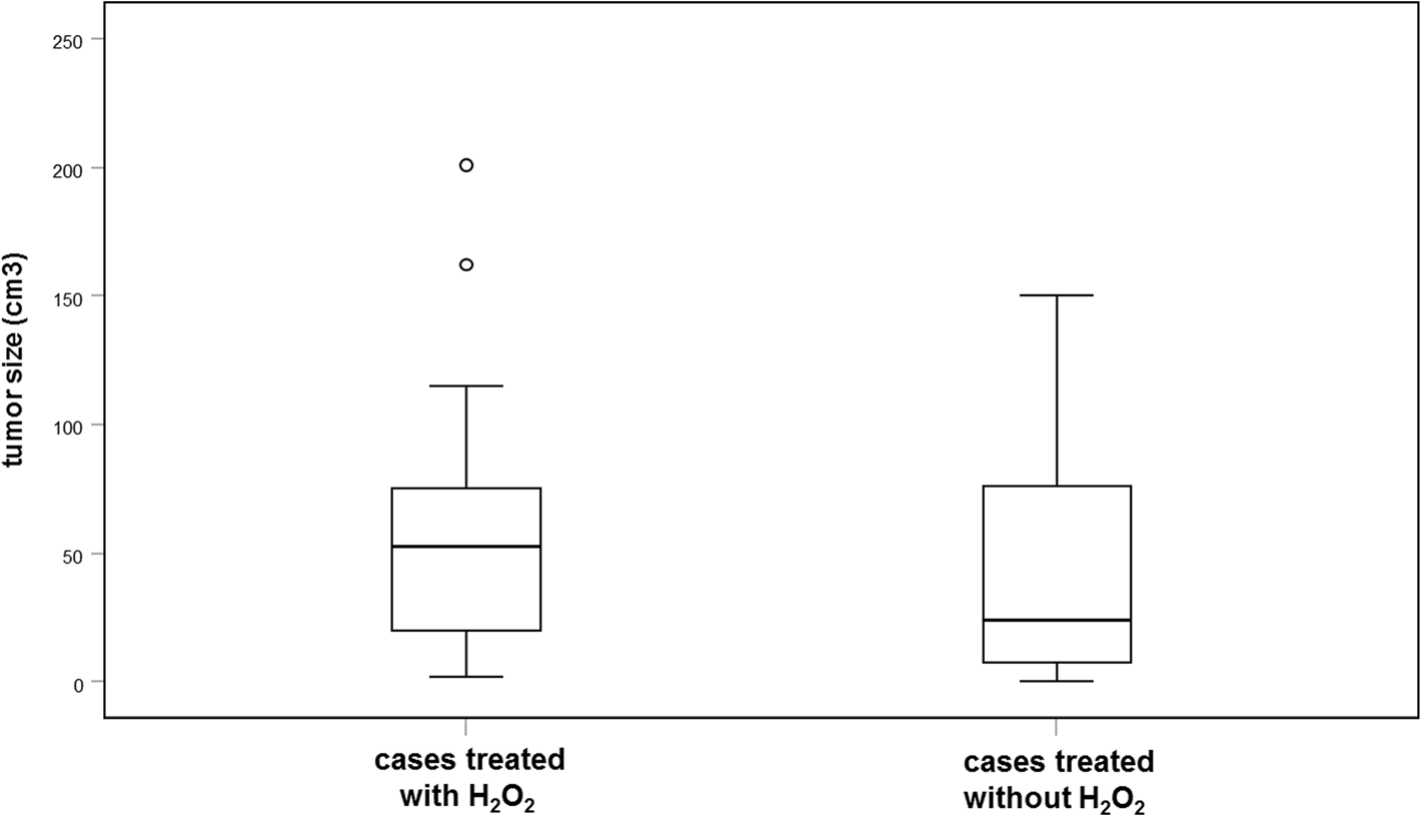
Box plots with preoperative tumor sizes in cubic centimeter. Size differences between cases treated with or without hydrogen peroxide were not significant

Radiological follow-up with X-rays and MRIs was performed regularly after surgery with intervals between 3 and 12 months. Images were evaluated by a musculoskeletal radiologist subspecialized in orthopedic oncology. Tumors were Campanacci grade III (*n* = 27) or grade II (*n* = 24). Mean size of all tumors was 52.0 (± 47.8) cm^3^. Those tumors treated with hydrogen peroxide were slightly larger, but this was not statistically significant (Table 1; Fig. 2). Size and intraoperative blood loss had a strong positive correlation (*r* = 0.501, *p* < 0.001).

**Table 1:** Treatment strategies with tumor characteristics and surgical and clinical parameter including the Musculoskeletal Tumor Society (MSTS) score at final follow-up. Mean (± standard deviation) and median (range) are depicted

	Lesion size at surgery in cm3	Number of recurrences	Satisfaction (0–10)	Disability (0–3)	MSTS-score (0–30)
Total (*n* = 51)	52 (± 48)	21	9 (4–10)	0 (0–2)	28 (15–30)
Curettage with H_2_O_2_ (*n* = 27)	58 (± 50)	6	9 (4–10)	0 (0–2)	28 (16–30)
Curettage without H_2_O_2_ (*n* = 24)	46 (± 46)	15	9 (5–10)	0 (0–2)	27 (15–30)

Until the final follow-up, no metastases were found.

Histological evaluation included information on angioinvasion and whether mitosis rate of the mononuclear cell population was high (≥ 20 mitotic figures in 10 different high-power (400×) microscopic fields). Angioinvasion was histologically verified in eight cases. High mitosis rate was found in four cases, from those, three had recurrences.

Clinical outcome was evaluated at the final follow-up using a standardized questionnaire. Visual analog scale ratings for patient satisfaction from 0 to 10 and limitations of musculoskeletal function of the affected body part were assessed. To compare limitations of musculoskeletal function of different body parts, score results from the Oxford Knee Score, Oxford Hip Score, Foot And Ankle Disability Index, and Quick Disabilities Of Arm Shoulder And Hand Score were grouped into four categories to rate disability (0 points = excellent function without disability to 3 points = highest disability). Overall clinical outcome was additionally evaluated by the Musculoskeletal Tumor Society (MSTS) score (Table 1) including functional parameter, emotional acceptance, and pain [24].

### Hydrogen peroxide use

Intralesional resection was done by vigorous curettage, additional burring, and further cleaning of the cavity with either physiologic salt solution or 3% hydrogen peroxide solution (988 mM, Hedinger, Stuttgart, Germany). Concise information on whether hydrogen peroxide was used or not was available for all cases. Before the tumor cavity was filled, it was repetitively flushed with the solution and cancellous bone margins were mechanically cleaned with a soaked compress. Mean application time of the solution was 3 min.

### In vitro analyses of hydrogen peroxide effects on GCT cells

The influence of hydrogen peroxide on cell viability and apoptosis induction was additionally quantified in vitro using five primary GCT-derived stromal cell lines isolated from fresh tumor tissue samples. Tumor tissue was mechanically cut in small pieces and digested with 1.5 mg/ml collagenase B (Roche Diagnostics, Mannheim, Germany) for 1 h at 37 °C in Dulbecco’s modified Eagle’s medium (DMEM) (Sigma-Aldrich, Taufkirchen, Germany) containing 4.5 g/l glucose, 10% fetal calf serum (Biochrom, Berlin, Germany), and 100 U/ml penicillin/streptomycin (Sigma-Aldrich). Cells were collected by centrifugation, washed twice in PBS, and cultured in DMEM. Twenty-four hours after plating, cells were carefully treated with Trypsin/EDTA (Sigma-Aldrich) leaving the giant cells attached in the culture flask. Detached neoplastic stromal cells were cultured for further two passages. The obtained GCT stromal cells were used for the investigation of hydrogen peroxide-induced effects. Cells were seeded in 24-well plates (50000 cells/well) and cultured in DMEM with or without the addition of hydrogen peroxide_._ The experiments were adapted to the clinical scenario simulating intraoperative flushing and cleaning with short time administration of hydrogen peroxide. Therefore, DMEM was removed from the well plates to flush the cells with 250 μl of highly concentrated (988 mM) ready to use 3% hydrogen peroxide solution (Hedinger, Stuttgart, Germany) which was equally used at surgeries. The hydrogen peroxide solution was kept in the well plates for 3 s, 1 min, or 3 min and then replaced by 1000 μl of DMEM to be further incubated for 24 h in DMEM without hydrogen peroxide to allow cell recovery.

Additional analysis of low-dose but long-time hydrogen peroxide treatment was performed with 0.5 mM, 1 mM, and 2 mM hydrogen peroxide solution for 4 h.

For the analysis of cell viability and apoptosis induction, adherent and detached cells were collected and the amount of viable cells was measured by propidium iodide staining (2.5 μg/ml) (Invitrogen, Karlsruhe, Germany) and subsequent flow cytometric quantification of positive cells as previously described [25]. Induction of apoptosis was quantified using the caspase-3 substrate NucView-488 (Linaris GmbH, Dossenheim, Germany) according to the manufacturer’s protocol with quantification of apoptotic cells by flow cytometry.

### Statistical analyses

Student’s *t* tests, analysis of variance (ANOVA) with post hoc testing, and Mann-Whitney *U* tests were used to compare differences depending on scale level and distribution of the data. Recurrence rates were analyzed by chi-square tests with likelihood ratios. Cumulative recurrence-free survival was evaluated by Kaplan-Meier analysis with log-rank testing to determine significant differences. Multivariate Cox regression analysis was additionally performed to determine significant risk factors for tumor recurrence in association with multiple factors. Statistical significance was assumed at a *p* value ≤ 0.05.

## Results

### Recurrences

From all 51 patients, we found later recurrences in 21 cases (41%). From 39 patients with first surgery at our institution, 11 (28%) developed recurrences.

Univariate analysis revealed that recurrence rate was not significantly different in tumors with angioinvasion, soft tissue infiltration, higher Campanacci grade, or larger size. Filling with cancellous bone instead of bone cement increased recurrence rate (2.3-fold; likelihood ratio 5.1, *p* = 0.024; *n* = 51). Higher recurrence rates were also observed if osteosynthesis was avoided (2.1-fold; likelihood ratio 4.5, *p* = 0.034; *n* = 51). There was a trend towards higher recurrence rate, if a pathological fracture was evident before surgery (1.9-fold; likelihood ratio 2.1, *p* = 0.149; *n* = 40) and if mitosis rate was high (2.0-fold; likelihood ratio 2.1, *p* = 0.147; *n* = 49).

**Fig. 3:**
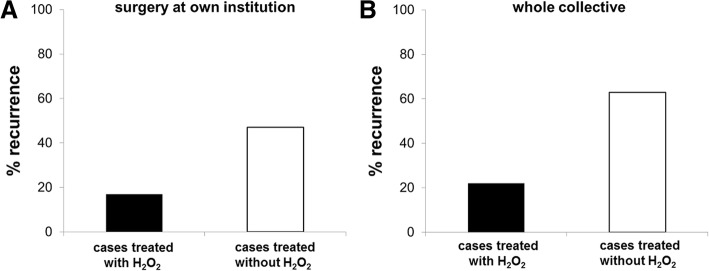
Recurrence rates depending on whether hydrogen peroxide (H_2_O_2_) was used or not to clean the tumor cavity after curettage: Significantly less recurrences were found after hydrogen peroxide treatment in the group of patients with surgery at our institution (**a**; *n* = 39) and in the whole collective (**b**; *n* = 51)

After hydrogen peroxide treatment, a strong and statistically significant impact on recurrence rate was observed (3-fold lower recurrence rates; details are depicted in Table 2 and Fig. 3).

**Table 2:** Recurrence rates with likelihood ratios and cumulative recurrence-free survival (RFS) rates at 2 years follow-up (2YFU), 5 years follow-up (5YFU), and 10 years follow-up (10YFU). Results are depicted for the whole collective and for cases with surgery at own institution (in domo) and surgery elsewhere (ex domo). Statistically significant hydrogen peroxide effects were found for all cases treated in domo and for the whole collective

	*n*	Recurrences (recurrence rate)	Likelihood ratio of recurrence (chi-square test)	2YFU-RFS (standard error)	5YFU-RFS (standard error)	10YFU-RFS (standard error)	Log-rank test for differences in RFS
Whole collective	**51**	**21 (0.41)**		**0.69 (0.07)**	**0.54 (0.08)**	**0.54 (0.08)**	
Surgery in domo	39	11 (0.28)	11.9 (*p* = 0.001)	0.72 (0.08)	0.68 (0.08)	0.68 (0.08)	*p* = 0.001
Surgery ex domo	12	10 (0.83)		0.42 (0.14)	0.17 (0.11)	0.17 (0.11)	
With H_2_O_2_ (in domo)	24	4 (0.17)	4.1 (*p* = 0.04)	0.86 (0.07)	0.79 (0.10)	0.79 (0.10)	*p* = 0.02
Without H_2_O_2_ (in domo)	15	7 (0.47)		0.48 (0.14)	0.48 (0.14)	0.48 (0.14)	
With H_2_O_2_ (whole collective)	27	6 (0.22)	8.8 (*p* = 0.004)	0.80 (0.08)	0.74 (0.09)	0.74 (0.09)	*p* = 0.002
Without H_2_O_2_ (whole collective)	24	15 (0.63)		0.47 (0.11)	0.31 (0.10)	0.31 (0.10)

Kaplan-Meier analysis was performed to estimate cumulative recurrence-free survival. After additional osteosynthesis, cumulative recurrence-free survival was significantly longer (at 2 years follow-up 0.75 (standard error (SE) 0.09) versus 0.54 (SE 0.10); at 10 years follow-up 0.67 (SE 0.11) versus 0.41 (SE 0.10); *p* = 0.05; *n* = 51). If mitosis rate was high, cumulative recurrence-free survival was significantly lower (at 2 years follow-up 0.25 (SE 0.22) versus 0.69 (SE 0.07); at 10 years follow-up: no case left versus 0.57 (SE 0.08); *p* = 0.019; *n* = 49) There was a trend for lower cumulative recurrence-free survival in case of a pathological fracture (at 2 years follow-up 0.38 (SE 0.17) versus 0.79 (SE 0.08); at 10 years follow-up 0.38 (SE 0.17) versus 0.65 (SE 0.10); *p* = 0.077; *n* = 40) and if cancellous bone was used instead of bone cement (at 2 years follow-up 0.50 (SE 0.20) versus 0.67 (SE 0.07); at 10 years follow-up 0.17 (SE 0.15) versus 0.60 (SE 0.08); *p* = 0.125; *n* = 51).

**Fig. 4:**
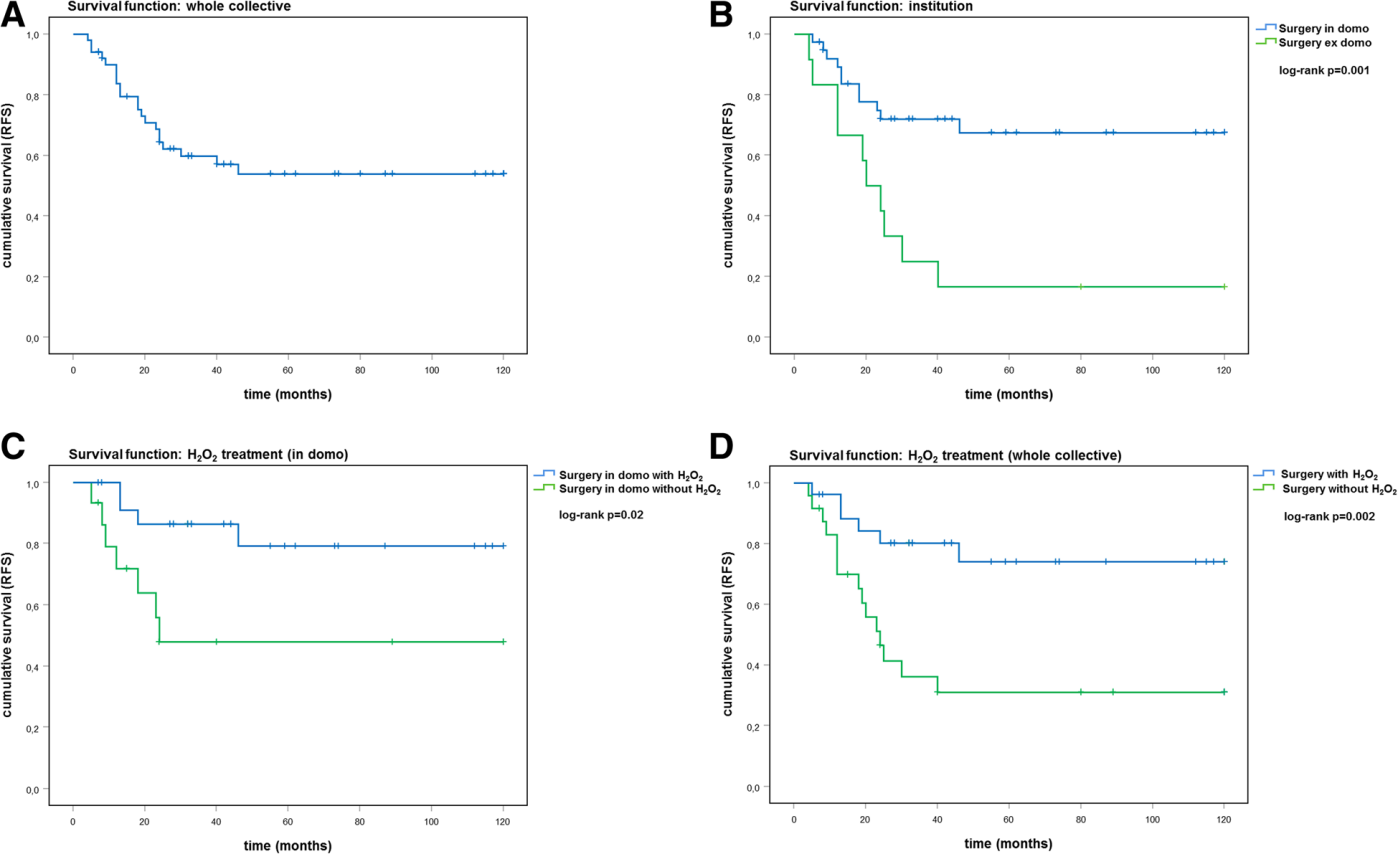
Kaplan-Meier analysis of cumulative recurrence-free survival (RFS). Survival curves are presented for the whole collective (**a**), for cases with surgery at our institution (in domo) versus surgery elsewhere (ex domo) (**b**), and for treatment with or without hydrogen peroxide (H_2_O_2_) in domo (**c**) and for the whole collective (**d**)

Kaplan-Meier analysis confirmed the strong positive effect of hydrogen peroxide treatment (details are depicted in Table 2 and Fig. 4).

Multivariate Cox regression analysis was performed to further determine risk factors for tumor recurrence. Hydrogen peroxide, bone cement, osteosynthesis, mitosis rate, and pathological fracture were included. Significantly higher risk for recurrence was confirmed for pathological fracture (hazard ratio 3.7; 95% confidence interval (CI) 1.1–12.7; *p* = 0.04), high mitosis rate (hazard ratio 15.6; 95% CI 2.0–124.6; *p* = 0.01), and lack of hydrogen peroxide use (hazard ratio 6.0; 95% CI 1.3–28.3; *p* = 0.02).

### Complications

Complication rate was 5 out of 51 and included one peripheral venous thrombosis and four infections.

### Clinical outcome

Hydrogen peroxide use versus no hydrogen peroxide use did not significantly influence functional and emotional outcome (compare Table 1).

### In vitro analyses of hydrogen peroxide effects on GCT cells

**Fig. 5:**
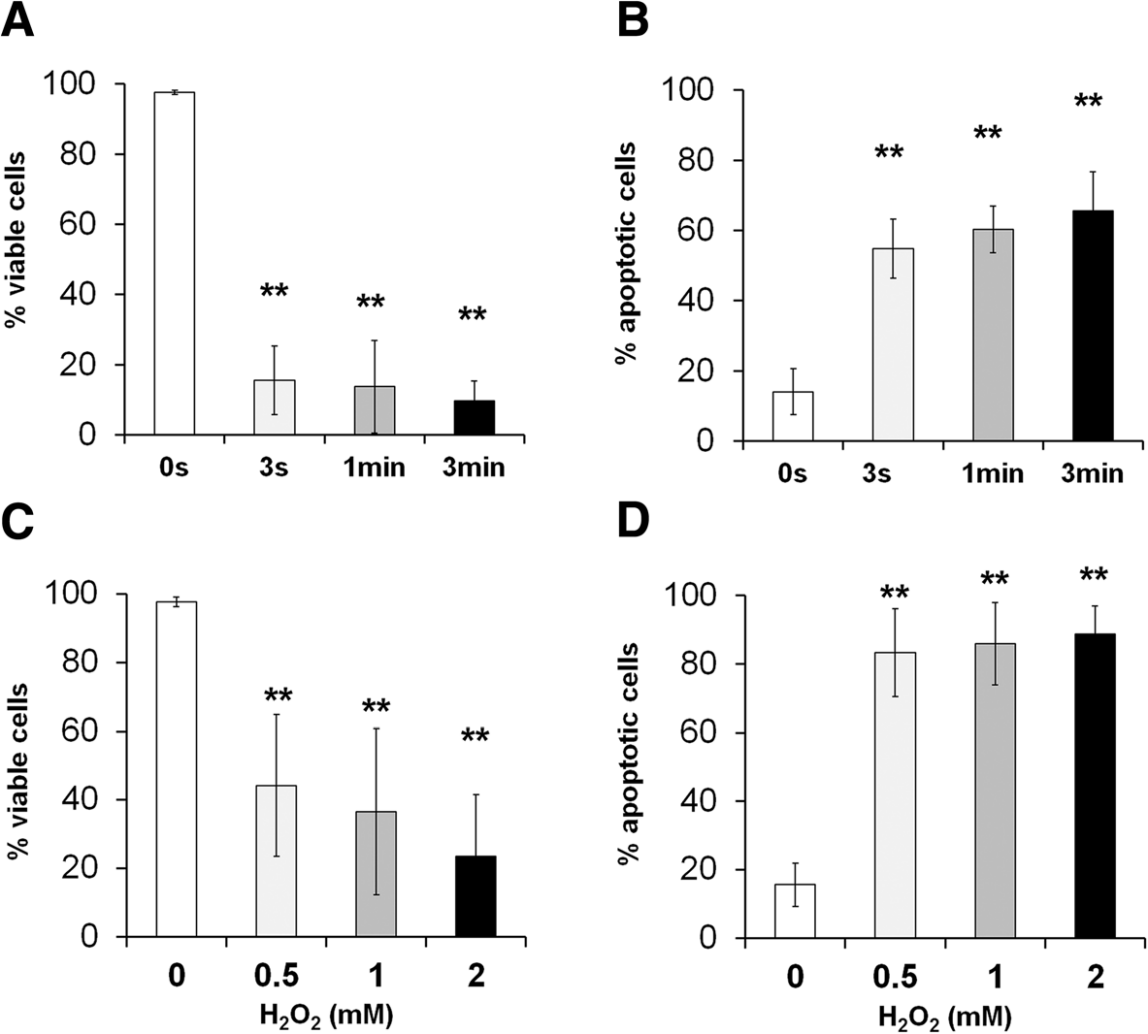
Analysis of cell viability (**a**) and apoptosis induction (**b**) after high-dose short-time hydrogen peroxide treatment of *n* = 5 primary GCT-derived stromal cell lines. Low-dose long-time treatment showed equal effects on cell viability (**c**) and apoptosis induction (**d**) (**p < 0.01)

Short-time treatment with highly concentrated (988 mM) ready to use 3% hydrogen peroxide solution simulated intraoperative treatment in the clinical situation and quickly induced GCT cell death. Quantification of caspase-3 activation, a key event within the apoptotic cascade, verified the apoptotic nature of hydrogen peroxide-induced cell death (Fig. 5a, b). Longer incubation time (4 h) with lower concentrations up to 0.5 mM produced comparable results (Fig. 5c, d).

## Discussion

The present series analyzed 51 advanced GCT cases of the upper and lower extremities with a majority of Campanacci grade III tumors. Contrary to other studies, which could not prove the additional effect of hydrogen peroxide due to its heterogeneous use, we for the first time could demonstrate that additional adjuvant local hydrogen peroxide application after curettage significantly reduced recurrence rate, increased cumulative recurrence-free survival, and was a significant risk factor in multivariate analysis. Further in vitro analysis confirmed hydrogen peroxide-induced apoptotic cell death of GCT cells as the underlying mechanism.

The literature describes heterogeneous use of local adjuvants to further clean the GCT cavity and kill potentially remaining GCT cells before filling the defect with bone cement or cancellous bone [5, 18, 22, 23]. Phenol was mainly used but is abandoned due to serious systemic toxicity and carcinogenic potential also for the surgeon by inhalation. Multiple other adjuvants have been described with, however, limited evidence due to rare and heterogeneous usage. Besides the filling with bone cement itself, local adjuvants can be divided into thermal adjuvants (cryotherapy with liquid nitrogen; cauterization) and toxic chemical substances such as alcohol, iodine, cyclophosphamide, phenol, and hydrogen peroxide [5, 18, 22, 23]. Hydrogen peroxide is a cheap and easily available adjunct which has been reported as an alternative for phenol with proven in vitro effects against GCT cells [26, 27], but its clinical value has not been proven so far [5, 18, 22, 23]. The present study documents statistically significant clinical effects of hydrogen peroxide.

In the present study, we for the first time tried to transfer the clinical intraoperative scenario of high-dose short-time hydrogen peroxide application also to in vitro analyses. Hydrogen peroxide is expected to kill potentially remaining tumor cells in the tumor cavity after aggressive curettage. These tumor cells are not macroscopically visible but potentially scattered and attached at the surface of the cavity. Hence, in vitro-isolated GCT cells attached at the well plate might be an appropriate target to test whether short-time application of highly concentrated hydrogen peroxide solution can diminish viable tumor cells in order to prevent later recurrence. After initial short-time treatment of the attached cells with hydrogen peroxide, cells were further incubated in DMEM for 24 h without hydrogen peroxide to allow recovery. This may resemble potential later tumor cell recovery after hydrogen peroxide is diluted and removed from the tumor cavity. Apart from general limitations based on the artificial setup of in vitro studies, our data support detrimental hydrogen peroxide effects on GCT cells. We found reduced cell viability and activated apoptotic pathways both after high-dose short-time application and after longer incubation times with lower hydrogen peroxide concentrations.

A further clinical advantage of hydrogen peroxide use was improved cleaning of the cancellous bone margins with better visibility compared to only cleaning with salt solution. Hence, the surgeon may detect and resect residual tumor tissue more easily. As a result of the present study, we generally recommend adjuvant local use of easily available hydrogen peroxide to finally clean the tumor cavity before filling the defect.

In agreement with most other studies [5, 16–19, 21], our data also support that bone cement filling reduces recurrence rate compared to cancellous bone filling, which can be explained by heat destruction of potentially remaining tumor cells during the exothermic polymerization of polymethylmethacrylate. Another advantage is better radiological identification of potential recurrences after bone cement filling [5]. The beneficial effect of bone cement filling, however, was not confirmed by some other studies [15, 22, 23]. The majority of our cases (45) had bone cement fillings, whereas only six cases had cancellous bone fillings, which limits statistical analysis. Statistical significance was only achieved for the recurrence rate analyzed by chi-square tests. Differences in cumulative recurrence-free survival did not reach statistical significance.

Age, gender distribution, localizations, and recurrence rates of our series are compatible with the literature. Our overall high recurrence rate can be explained by the majority of initially complicated and advanced cases in our university institution, specialized in orthopedic oncology, as well as by the cases initially treated in other surrounding hospitals and referred to us for further follow-up due to their complexity. Another reason will be our philosophy of avoiding extralesional wide resection with endoprosthetic reconstruction in the young GCT population as much as possible and even in cases with partial joint-line infiltration, due to long-time hazards related to a tumor mega prosthesis. Some authors, however, prefer wide resection in advanced Campanacci grade III tumors [4] to minimize recurrences and avoid multiple surgeries [5, 16]. Improvements of survival and function of today’s tumor endoprostheses need further long-time analysis.

Histologically, osteoclast-like multinuclear giant cells and mononuclear neoplastic stroma cells influence tumor activity [1, 28], but histological classification of GCT was not reliable in predicting prognosis [5, 15, 29]. We also evaluated mitosis rate of the mononuclear cell population and found that higher mitosis rate was associated with significantly lower recurrence-free survival and higher hazard ratio in multivariate analysis, which is compatible with another report [21].

Molecular pathogenesis of GCT [30, 31] will influence future therapeutic strategies. Adjuvant or neo-adjuvant systemic treatment with denosumab, a RANKL inhibitor, is increasingly used for cases where surgical resection would cause inappropriate morbidity [32]. The present study did not analyze denosumab treatment which might improve outcome. From the authors’ experience, however, denosumab will be more appropriate for advanced tumors in the pelvis and spine where complete surgical tumor removal is not possible without mutilation. Even higher postoperative recurrence rates in cases with neo-adjuvant denosumab treatment have been reported [33], which might be related to tissue remodeling with less obvious tumor margins after denosumab treatment making complete tumor removal more difficult for the surgeon. These effects, however, are controversial as beneficial reduction of tumor size was reported after denosumab treatment [34] and randomized trials have been initiated for further evaluation [8]. Optimal duration of denosumab treatment also remains unclear as well as the risk for the induction of secondary osteosarcomas [7, 8, 33, 34].

We observed higher recurrence rates and lower cumulative recurrence-free survival in cases, where osteosynthesis was avoided. A possible explanation might be more vigorous curettage and improved visibility in cases with additional stabilization via osteosynthesis, as the surgeon might have been less concerned about intra- or postoperative fracture.

Important limitations of the present study have to be acknowledged. Compared to some other studies, absolute numbers of cases were low and depending on the different parameter, data was not available for all cases. Analysis was retrospectively performed and hydrogen peroxide was heterogeneously used with potential bias. As an additional cell-toxic adjunct will be more likely used in complicated cases, the beneficial effect on recurrence rate will be, however, rather underestimated than overestimated. For final evaluation, further studies are needed with higher patient numbers, potentially prospective study design and specific selection criteria for hydrogen peroxide use. Nevertheless, the strength of the present study is proof of principle for hydrogen peroxide effectiveness by both in vitro cell culture studies and clinical analyses with sufficient numbers of equally treated patients with and without hydrogen peroxide use.

## Conclusion

For the first time, this study proves the clinical effectiveness of adjuvant local hydrogen peroxide to clean the cavity before defect filling. Hydrogen peroxide treatment significantly reduced recurrence rate and increased cumulative recurrence-free survival. Reduction of viable GCT cells by induction of apoptosis analyzed in vitro supports this.

## Abbreviations

DMEM: Dulbecco’s modified Eagle’s medium
EDTA: Ethylenediaminetetraacetic acid
GCT: Giant cell tumor
H_2_O_2_: Hydrogen peroxide
MSTS-score: Musculoskeletal Tumor Society score
PBS: Phosphate-buffered saline
RFS: Recurrence-free survival
YFU: Years follow-up
